# Scalable physical activity intervention for youth with disability: Burn 2 Learn adapted cluster randomized controlled trial

**DOI:** 10.1186/s12966-025-01829-1

**Published:** 2025-10-09

**Authors:** David R. Lubans, Nora Shields, Narelle Eather, Jordan J. Smith, Michael Noetel, Charles H. Hillman, Chris Lonsdale, Christopher Oldmeadow, Ashleigh Stuart, Sarah G. Kennedy, James Boyer, Pierre Comis, Laura Roche, Taren Sanders, Tara Finn, Angus A. Leahy

**Affiliations:** 1 Global Sport and Movement Collaborative, University of Newcastle, Callaghan, NSW Australia; 2 Hunter Medical Research Institute, New Lambton Heights, New South Wales, Australia; 3 Faculty of Sport and Health Sciences, University of Jyväskylä, Jyväskylä, Finland; 4 Olga Tennison Autism Research Centre, La Trobe University, Melbourne, VIC Australia; 5 School of Psychology, University of Queensland, Queensland, QLD Australia; 6 Department of Physical Therapy, Movement and Rehabilitation Sciences, Northeastern University, Boston, MA USA; 7 Department of Psychology, Northeastern University, Boston, MA USA; 8 Institute for Positive Psychology and Education, Australian Catholic University, North Sydney, New South Wales, Australia; 9 School of Medicine and Public Health, College of Health, Medicine and Wellbeing, University of Newcastle, Callaghan, NSW Australia; 10 Translational Health Research Institute, Western Sydney University, Darug Country, Richmond, NSW Australia; 11 New South Wales Department of Education, Turrella, New South Wales Australia; 12 Special Olympics Australia, Callaghan, Concord West, New South Wales Australia

## Abstract

**Background:**

Youth with disability are less physically active and more likely to have chronic health conditions than their peers without disability. The aim of our study was to assess the effectiveness of a scalable school-based physical activity intervention for youth with disability on functional capacity and a range of secondary outcomes.

**Methods:**

We conducted a two-arm cluster randomized controlled trial involving adolescents aged 15–19 years with diagnosed disabilities (*N* = 255) from 28 secondary schools in New South Wales, Australia. Schools were randomized to the Burn 2 Learn adapted (B2La) intervention, or a wait-list control. The B2La intervention included foundational resistance exercises (e.g., push-ups, bodyweight squats), aerobic exercises (e.g., shuttle runs), and sport skills (e.g., catching, kicking), delivered as classroom activity breaks 2–3 times per week by trained special education teachers. The primary outcome was functional capacity assessed using the 6-min walk or push test. Secondary outcomes were muscular fitness, body mass index, physical activity (accelerometers), resistance training motor competence, motivation for physical activity, high-intensity interval training self-efficacy, quality of life, and externalizing behaviors. Assessments were conducted at baseline, 6-months (primary endpoint), and 9-months (follow-up).

**Results:**

At 6-months, the intervention group demonstrated a significant improvement in functional capacity, with a group-by-time effect of 20.3 m (95% CI, 3.1–37.1). At 9-months, the effect was 17.8 m (95% CI, 0.0–35.6). The intervention had a small effect on muscular fitness, resistance training motor competence, and high-intensity interval training self-efficacy. No effects were observed for the other outcomes and no adverse events were recorded.

**Conclusions:**

Physical activity breaks delivered by special education teachers during the school day improved functional capacity and a range of secondary outcomes in youth with disability. Activity breaks may need to be longer, more frequent, or more intense to achieve clinically important health effects.

**Trial registration:**

Australian New Zealand Clinical Trials Registry Number: ACTRN12621000884808; prospectively registered 15th November, 2021.

**Supplementary Information:**

The online version contains supplementary material available at 10.1186/s12966-025-01829-1.

## Introduction

Individuals with disability face widespread barriers to accessing health services and have poorer health outcomes than those without disability [1]. The World Health Organization’s Global Disability Action Plan reports the burden of disability can be reduced by addressing the determinants of health, including physical activity [1]. Unfortunately, individuals with disability are up to 62% less likely to meet physical activity guidelines [2] and more likely to have co-occurring chronic lifestyle diseases [3, 4] than those without disability. As behaviors established during adolescence track into adulthood [5], there is an urgent need to identify scalable interventions to improve health outcomes and alter physical activity trajectories among youth with disability.

Physical activity interventions targeting youth with disability have yielded mixed results [6–9]. This might be expected given the barriers to physical activity participation among youth with disability and the heterogenous profiles and severities [10, 11]. For instance, Kapsal and colleagues [7] found such interventions produce moderate-to-large improvements in physical and psychosocial health among youth with intellectual disabilities. However, attempts to change physical activity behavior in youth with disability have produced modest effects [6]. A recent review by Lai et al. [12] highlighted the methodological limitations of previous interventions and identified three key priorities: (i) develop more precise interventions to enable replication, (ii) identify strategies to achieve sustainable changes in physical activity, and (iii) create scalable interventions that can be implemented across various settings and among youth with diverse disabilities. The final point is particularly important, as previous interventions have typically been small-scale and conducted in clinical settings with one specific group.

Schools are ideal settings for delivering ‘scalable’ physical activity interventions for youth, including those with disability [13]. For interventions to be ‘scalable’ they need to be effective, adaptable, and designed to address barriers to implementation in schools, such as lack of resources (e.g., time, equipment), skills (e.g., teacher competence), and support from education departments (i.e., physical activity is not a priority) [14, 15]. Physical activity breaks are a scalable intervention that have been used in elementary schools for decades [16]. Activity breaks are rarely used in secondary schools [17] and represent a novel strategy to increase physical activity among youth with disability during school hours and may also support the development of physical literacy (i.e., motivation, confidence, physical competence and knowledge to be physically active across the lifespan) [18].

We developed Burn 2 Learn (B2L) [19, 20], a teacher-facilitated activity break intervention for older adolescents in mainstream schools. The B2L sessions, integrated into classroom lessons, enhanced students’ cardiorespiratory and muscular fitness, improved mental health among at-risk students [19], and benefited classroom engagement [19, 20]. We were approached by a local school to adapt the intervention for youth with disability. After conducting a successful pilot study in one secondary school [21], we partnered with the New South Wales Department of Education and Special Olympics Australia to design and conduct an effectiveness trial [22]. Our primary aim was to determine the effect of the Burn 2 Learn adapted (B2La) intervention on functional capacity (primary outcome) in youth with disability. We selected the 6-min walk or push test as the primary outcome measure for our study due to the following reasons. First, it has demonstrated good reliability in adolescents with disability (ICC = 0.82) [23]. Second, it was expected to be responsive to our intervention, which combined shuttle runs with resistance exercise and sports skills. Third, the 6-min walk test (or the 6-min push test for wheelchair users) could be administered to all participants, ensuring inclusivity across disability groups. Finally, although adolescent data are limited, evidence from adults with chronic health conditions indicates that relatively small improvements in the 6-min walk test (24 to 44 m) are considered clinically meaningful [24]. Secondary aims of our study were to assess the impact of the B2La intervention on participants’ physical activity, muscular fitness, body composition, quality of life, physical literacy, and externalizing behaviors. We also conducted a process evaluation to determine program acceptability, implementation, and sustainability.

## Methods

### Study design

Our trial protocol has been described in detail previously [22]. Briefly, the trial was registered with the Australian New Zealand Clinical Trials Registry (ACTRN12621000884808) and is reported according to the CONSORT guidelines extended to cluster randomized controlled trials (RCTs) [25]. We evaluated B2La using a two-arm parallel group cluster RCT with a treatment group and wait-list control group. The RCT was conducted in two waves (Supplementary Fig. 1). The first in 2022 (10 schools) and the second in 2023 (18 schools). We conducted assessments at baseline, 6- (primary endpoint) and 9-months from baseline (secondary endpoint). Baseline data collection occurred in the school term preceding intervention delivery (i.e., Term 1 [February to April]). Data collection (i.e., 6-months) occurred at the end of Term 3 (August to September) and follow-up assessments (i.e., 9-months) were completed in Term 4 (November to December).

### School recruitment and participants

All Government, Catholic, and Independent secondary schools with student cohorts including older adolescents living with disability were eligible to participate. We recruited two special education teachers per school, who facilitated intervention delivery. Students were eligible to participate if they were: (i) in Grades 10 to 12 (15–19 years) and had a disability (including neuro-developmental, physical, behavioral, emotional, intellectual, and sensory disabilities), (ii) able to follow simple verbal instructions in English, and (iii) able to participate in vigorous intensity exercise (wheelchair users were eligible). School principals, teachers, parents, and students all provided informed written consent prior to enrolment.

### Sample size and power calculation

Power calculations were based on the primary outcome of functional capacity, assessed using the 6-min walk or push test [23, 24]. We estimated a treatment effect of 80 m would represent a minimal clinically important difference (MCID) in our population. Through simulations (*n* = 10,000) and using data from our pilot study (i.e., baseline post-test correlation of* r* = 0.60, standard deviation of 90 m and intraclass correlation of 0.2), we determined 30 schools with 7 participants per school would be sufficient to detect a MCID of 80 m with 90% power at a 5% significance level. Allowing for 30% loss to follow-up at 6-months, we aimed to recruit 10 students from 30 schools (*N* = 300).

### Randomization

To ensure balance across groups, pairs of school were matched on school type (i.e., mainstream school support class or special education school), school sector (i.e., government or other), geographic location, and school socioeconomic status. Pairs of schools were then randomized to the intervention or wait-list control conditions using a random number producing algorithm by an independent statistical analysis service after baseline data collection.

### Intervention

Our protocol paper provides a detailed description of the B2La intervention [22]. Special Education teachers attended a full-day professional development workshop delivered by the research team. Briefly, the workshop provided teachers with the training and resources needed to facilitate the school-based B2La sessions, and involved a combination of theoretical (e.g., program rationale, benefits of activity breaks, and school implementation plan) and practical (e.g., participation in a B2La session, peer assessment of exercise technique and overview of how to use program resources) activities. 

B2La sessions involved a combination of foundational resistance (i.e., push up, squat, front support, lunge) and aerobic (i.e., shuttle run, high knees, running on the spot) exercises and sport skills (e.g., catching, dribbling, throwing). The B2La intervention also included: (i) an information seminar for students, delivered by the teachers, (ii) goal setting activity booklet, (iii) access to the purpose-built B2L smartphone application and heart rate monitors, and (iv) parental support videos. Guided by the Consolidated Framework for Implementation Research [26] and our conceptual model for scaling-up HIIT programs for population health [27], we used a range of strategies to support intervention implementation (Supplementary Fig. 2).

The intervention was delivered in four phases. In Phases 1–3 (term 2–term 3; May–September 2022 and 2023), Special Education teachers were asked to facilitate two-to-three B2La sessions/week during scheduled ‘Learning Support Lessons’. This is a time when youth with disability are working separately to those without disability (for those attending mainstream schools). Phase 1 started with a 4-week block to familiarize the students with the B2La session structure and had a greater focus on developing students’ foundational exercise skill competence. In Phase 4 (term 4; October-December 2022 and 2023), students were encouraged to engage in B2La sessions outside of school, however, teachers were able to continue to facilitate school-based sessions if they wanted to.

The B2La sessions lasted 10–20 min, including a warm-up. Task complexity and variation within the B2La sessions increased progressively across the study period. Given the wide range of participants’ abilities, teachers were encouraged to adapt both exercises and task complexity to meet individual student needs. Some sessions required minimal cognitive demand, with students performing only two distinct exercises, while other sessions involved alternating between up to six unique exercises. Professional learning workshops supported teachers in considering the specific requirements of their students and identifying necessary adaptations. Additional equipment was provided to schools where required. For example, wheelchair users were provided with lightweight dumbbells to perform upper body resistance exercises in place of push-ups. Students were encouraged to achieve a target intensity of ≥ 80% of their age-predicted maximum heart rate.

### Measures and data collection

Assessments were conducted at schools by trained research assistants. Our intention was to blind assessors to group allocation. However, our checks revealed group allocation was revealed to assessors at three schools at follow-up. Questionnaires were completed with the assistance of research assistants using electronic tablets. Physical assessments were conducted by a research assistant of the same sex where possible. We collected demographic information (e.g., age, sex, ethnicity, country of birth, residential postcode, and parent/caregivers’ education level) at baseline. Due to physical and intellectual limitations, not all participants completed all measures (see Table 3).

### Primary outcome

#### Functional capacity

Our primary outcome was functional capacity, assessed using the 6-min walk test [28] or 6-min push test (for wheelchair users) [29].

### Secondary outcomes

#### Muscular fitness

Lower body muscular endurance was assessed using the 30 s sit-to-stand test [30]. We used a modified version of the 90-degree push-up test (performed on knees) to assess upper body muscular endurance [31].

#### Body mass index

Body weight and height were measured using portable digital scales (A&D Medical UC-352-BLE Digital Scales) and stadiometer (Seca 213 Portable Height Measuring Rod Stadiometer) respectively. Body mass index (BMI) was calculated using the standard formula (weight[kg]/height[m]^2^). Age- and sex-specific BMI z-scores were used to classify participants into weight categories using the International Obesity Task Force cut-offs [32].

#### Physical activity

Participants wore an ActiGraph GT9X Link accelerometer on their non-dominant wrist for seven consecutive days (3-days of 10-h minimum wear time), which is an acceptable measure for youth with disability [33]. We report steps/day and moderate-to-vigorous physical activity (i.e., mean minutes per day) on weekdays and weekend days. Existing thresholds [34] were used to classify the intensity of participants’ physical activity using the GGIR package [35].

#### Resistance training skill competence

Competence in four foundational resistance training skills (i.e., push-ups, lunge, squat, front support chest touch) was determined using video analysis [36]. Participants completed two trials consisting of four repetitions for each skill. Before performing each skill, participants observed a video demonstrating correct performance. For visually impaired students, verbal instructions were provided rather than video demonstrations. Exercise skills were assessed against predetermined performance criteria, ranging from 3–5 criteria per skill.

#### Motivation for physical activity

Autonomous motivation for physical activity was assessed using the identified and intrinsic subscales from the 'Behavioral Regulations in Exercise Questionnaire-2 [37].

#### Self-efficacy for high-intensity interval training

Confidence to participate in HIIT was assessed using the High-Intensity Interval Training Self-efficacy Questionnaire [38].

#### Externalizing behaviors

Teachers completed the conduct and hyperactivity sub-scales from the Strengths and Difficulties Questionnaire [39] for each participant.

#### Quality of life

Health-related quality of life (HRQoL) was assessed using the Child Health Utility 9-Dimensions [40].

### Process evaluation

We conducted a process evaluation to assess the dose, fidelity, acceptability, appropriateness, and sustainment of the intervention. Data sources included teacher logbooks, app usage analytics, session observations and teacher surveys (see Supplementary Table 2). Teachers reported any adverse events in their Handbook.

### Statistical analyses

 Statistical analyses were conducted by an independent statistician who was blinded to condition allocation, using linear mixed models and generalized linear mixed models with multinomial distributions and cumulative logit link functions in SAS V 9.4 (SAS Institute Inc, Cary, NC), with alpha levels set at *p* < 0.05. Models assessed the impact of group, time (treated as categorical), and the group-by-time interaction. Random intercepts were included for participants to account for repeated measures, and school to account for clustering. Covariance of the random effects was modelled as unstructured and parameterized through its Cholesky root. Fixed effects were estimated using Laplace’s method with degrees of freedom for linear models calculated using the containment method, while between-within approximation was used for multinomial models. Mixed models used a modified intention-to-treat approach. Analyses at 6-months included all participants who had an outcome measurement at baseline or 6-months, while analyses at 9-months included participants who had an outcome measurement at any baseline, 6-months, or 9-months. Sensitivity analyses using multiple imputation and complete cases were completed, with multiple imputation conducted using fully conditional specification, with predictive mean matching used for continuous variables and logistic regression for ordinal variables. Fifty imputed datasets were generated for each treatment group then the effect of the intervention estimated separately for each imputed dataset and pooled using Rubin’s rules. Four potential moderators were identified a priori (i.e., intellectual disability status, socio-economic status, sex, and weight status) and explored using interaction terms (i.e., time-by-treatment-by-moderator)- subgroup results are provided in the supplementary materials.

## Results

**Fig. 1:**
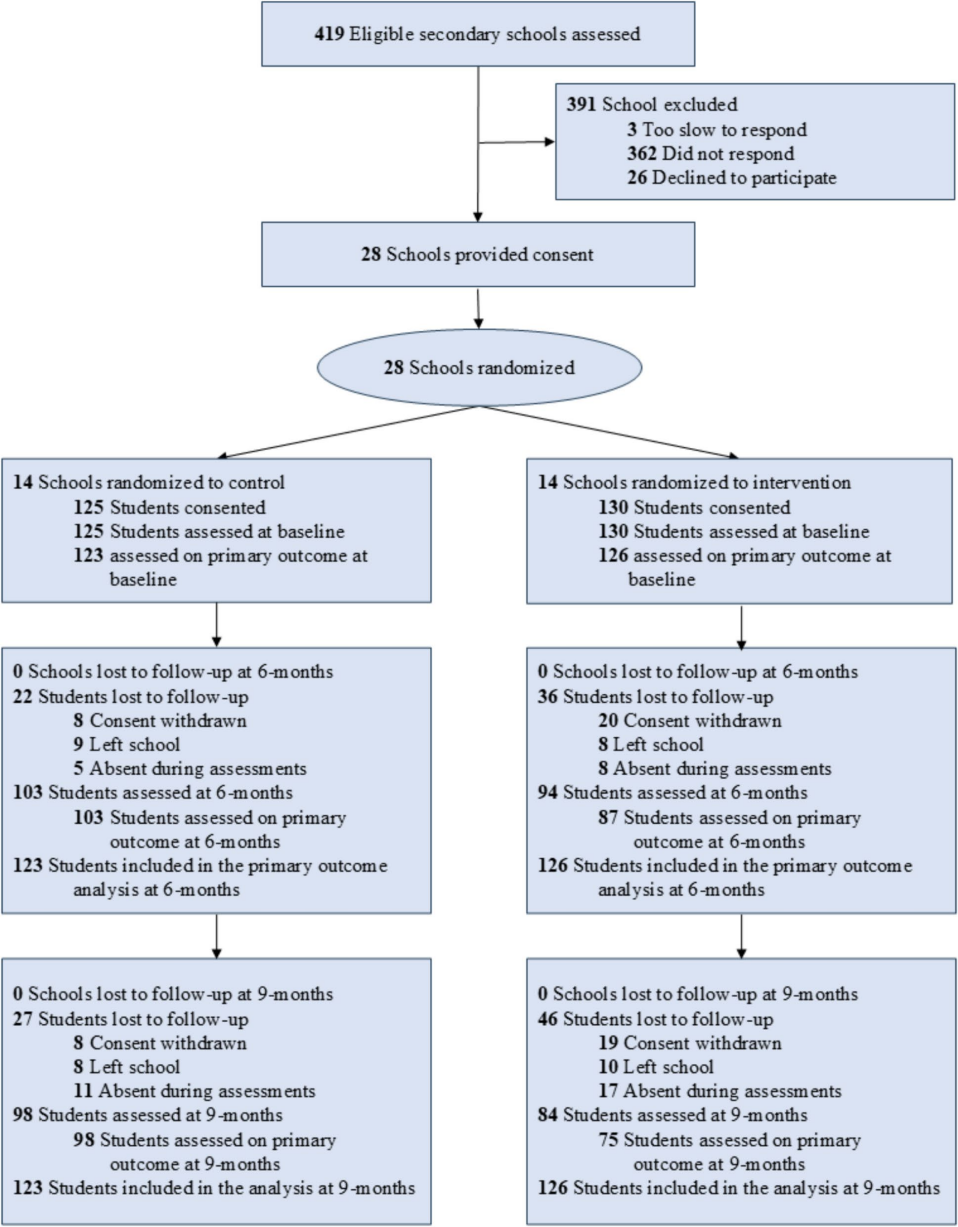
CONSORT flow diagram

Participant flow is reported in Fig. 1. We recruited 255 students (68 girls, mean age 15.7 ± 1.0 years from 28 schools.). Of these, 197 (77.3%) and 182 (71.4%) were assessed at 6-months (primary endpoint) and 9-months, respectively. Most participants had multiple disabilities (Table 1). More than half of participants had an intellectual disability (56.9%) or autism (52.5%). Most participants were born in Australia (97.6%) and spoke English at home (96.9%). Based on the International Obesity Task Force cut points, 41 (16.1%) participants were classified as overweight and 60 (23.5%) as obese. Baseline, 6- and 9-month and ICC values are reported in online Supplementary Table 1.

**Table 1:** Baseline characteristics of the study sample

Characteristics	Control (*n* = 125)	Intervention (*n* = 130)	Total (*n* = 255)
Age, mean (SD), y	15.9 (1.0)	15.5 (1.0)	15.7 (1.0)
Girls, n (%)	24 (19.2)	44 (33.8)	68 (26.7)
Born in Australia, n (%)^1^	121 (97.6)	125 (97.7)	246 (97.6)
English spoken at home, n (%)^1^	120 (96.0)	127 (97.7)	247 (96.9)
Cultural background, n (%)^1^			
Australian	90 (72.6)	107 (82.9)	197 (77.9)
European	8 (6.5)	6 (4.7)	14 (5.5)
African	3 (2.4)	2 (1.6)	5 (2.0)
Asian	6 (4.8)	3 (2.3)	9 (3.6)
Middle Eastern	1 (0.8)	0 (0.0)	1 (0.4)
Other	16 (12.9)	11 (8.5)	27 (10.7)
Indigenous descent, n (%)^1^			
Yes	23 (18.5)	25 (19.4)	48 (19.0)
No	101 (81.5)	104 (80.6)	205 (81.0)
Socioeconomic status, n (%)^1,2^			
Low	8 (6.6)	17 (14.3)	25 (10.4)
Medium	75 (62.0)	65 (54.6)	140 (58.3)
High	38 (31.4)	37 (31.1)	75 (31.3)
Weight status, n (%)^3^			
Underweight	14 (11.2)	15 (11.5)	29 (11.4)
Healthy weight	62 (49.6)	55 (42.3)	117 (45.9)
Overweight	17 (13.6)	24 (18.5)	41 (16.1)
Obese	28 (22.4)	32 (24.6)	60 (23.5)
Disability types, n (%)			
Autism spectrum disorder	78 (62.4)	56 (43.1)	134 (52.5)
Behavioral disability	7 (5.6)	10 (7.7)	17 (6.7)
Sensory disability	5 (4.0)	3 (2.3)	8 (3.1)
Emotional disturbance	8 (6.4)	12 (9.2)	20 (7.8)
Intellectual disability	69 (55.2)	76 (58.4)	145 (56.9)
Physical disability	6 (4.8)	15 (11.5)	21 (8.2)

^1^Missing data: country of birth (*n* = 3), language spoken at home (*n* = 3) cultural background (*n* = 2), indigenous descent (*n* = 2), socio-economic status (*n* = 15) and weight status (*n* = 8)

^2^Socioeconomic status was determined by population tertile using socio-economic indexes for areas of relative socioeconomic disadvantage based on residential postcode

### Change in primary outcome

We observed a small group-by-time effect (20.3 m, 95% CI 3.6 to 37.1) in favor of the intervention group (Table 2). Intervention effects did not differ by intellectual disability (*p* = 0.604), socio-economic status (*p* = 0.331), sex (*p* = 0.536), or weight status (*p* = 0.821). Improvements in functional capacity were sustained at 9-months (17.8 m, 95% CI 0.0 to 35.6).

**Table 2:** Changes in functional capacity at 6-months between participants randomized to usual practice or the B2La intervention

Model	No. of participants (clusters)		Mean change (SD) from baseline (meters)		Adjusted difference^4^ (meters)	
	**Control**	**Intervention**	**Control**	**Intervention**	**Estimate (95% CI)**	***p*-value**
Primary analysis (mixed model)^1^	123 (14)	126 (14)	−11.65 (54.03)	10.13 (63.45)	20.34 (3.62, 37.06)	0.017
Multiple imputation^2^	125 (14)	130 (14)	−10.12 (61.73)	10.05 (80.34)	20.18 (2.31, 38.05)	0.027
Complete case^3^	101 (14)	84 (14)	−11.65 (54.03)	10.13 (63.45)	21.78 (4.83, 38.73)	0.012

^1^Included all participants who completed the 6-minute walk test at baseline or 6 months

^2^Included all participants enrolled in the study

^3^Included all participants who completed the 6-minute walk test at baseline and 6 months

^4^Adjusted difference is the difference in change from baseline in treatment 2 compared to treatment 1 [(Intervention follow-up minus Intervention baseline)—(Control follow-up minus Control baseline)], adjusted for clustering

### Changes in secondary outcomes

The number of participants completing each measure at baseline 6- and 9-months is reported in Table 3. We observed a small improvement in lower body muscular endurance in favor of the intervention group at 6-months (0.8 repetitions, 95% CI 0.0 to 1.7), which was sustained at 9-months (1.0 repetitions, 95% CI 0.2 to 1.9) (Table 3). We observed a group-by-time effect for BMI in favor of the control group at 9-months (0.87 kg/m^2^, 95% CI, 0.21 to 1.53). Participants’ resistance training skill competence improved at 6-months and effects were sustained at 9-months. We observed a group-by-time effect for HIIT self-efficacy at 9-months [2.32 units, 95% CI, 1.09 to 4.95) (Table 4). 

The group-by-effect for hyperactivity was not statistically significant (0.52 units, 95% CI, 0.24 to 1.09). However, girls in the intervention group were 79% less likely to have a higher hyperactivity score at 6-months (compared to baseline), in comparison with those in the control group (0.21, 95% 0.05 to 0.95). Moderation effects and subgroup analyses are reported in Supplementary Tables 2 to 9.

**Table 3:** Changes in fitness, physical activity, and motor competence

**Secondary outcomes**		**Participants (clusters)**		**Mean change from baseline (SD)**		**Adjusted difference at follow-up**	
	**Time**	**Control**	**Intervention**	**Control**	**Intervention**	**Estimate (95% CI)**	***P* value**
Functional capacity, m	9 months	123 (14)	127 (14)	−12.27 (67.99)	8.30 (73.93)	17.83 (0.01, 35.64)^1^	**0.050**
Upper body muscular endurance, reps	6 months	117 (14)	115 (14)	0.93 (6.47)	1.36 (5.84)	1.42 (0.59, 3.42)^2^	0.413
Upper body muscular endurance, reps	9 months	120 (14)	116 (14)	0.73 (8.01)	2.18 (5.61)	1.76 (0.70, 4.43)^2^	0.223
Lower body muscular endurance, reps	6 months	122 (14)	126 (14)	−0.21 (3.12)	0.47 (2.59)	0.83 (0.00, 1.66)^1^	**0.050**
Lower body muscular endurance, reps	9 months	122 (14)	127 (14)	0.16 (3.31)	1.08 (2.60)	1.03 (0.15, 1.91)^1^	**0.022**
Body mass index, kg/m^2^	6 months	122 (14)	128 (14)	0.24 (1.19)	0.36 (1.34)	0.12 (−0.24, 0.48)^1^	0.516
Body mass index, kg/m^2^	9 months	123 (14)	128 (14)	0.00 (1.36)	0.98 (3.96)	0.87 (0.21, 1.53)^1^	**0.010**
MVPA, mins/weekday	6 months	74 (14)	83 (14)	6.27 (19.54)	−1.65 (21.59)	0.33 (0.10, 1.15)^2^	0.080
MVPA, mins/weekday	9 months	74 (14)	85 (14)	6.97 (14.86)	4.78 (20.84)	0.48 (0.12, 1.97)^2^	0.296
MVPA, mins/weekend day	6 months	52 (14)	62 (14)	3.84 (17.47)	4.84 (20.89)	0.95 (0.17, 5.32)^2^	0.949
MVPA, mins/weekend day	9 months	59 (14)	66 (14)	2.39 (21.87)	−0.91 (12.92)	0.45 (0.07, 2.82)^2^	0.381
Steps/weekday	6 months	80 (14)	80 (14)	−280 (2,897)	−250 (2,943)	−841 (−2,225, 543)^1^	0.229
Steps/weekday	9 months	80 (14)	82 (14)	1383 (2,645)	205 (3,945)	−1346 (−3,076, 383)^1^	0.126
Steps/weekend day	6 months	54 (14)	63 (14)	582 (3,325)	1,059 (2,598)	0.63 (0.16, 2.51)^2^	0.481
Steps/weekend day	9 months	60 (14)	66 (14)	1363 (3,761)	265 (3,947)	0.45 (0.10, 1.98)^2^	0.276
Resistance training skill competence, units	6 months	118 (14)	119 (14)	2.85 (4.44)	7.32 (5.10)	13.11 (5.26, 32.72)^2^	** <.001**
Resistance training skill competence, units	9 months	119 (14)	119 (14)	3.30 (4.51)	7.66 (5.60)	13.03 (5.25, 32.33)^2^	** <.001**

*MVPA* moderate-to-vigorous physical activity, *m* meters, *reps* repetitions, *SD* standard deviation, *95% CI* 95% confidence intervals

^1^Adjusted difference [(Intervention post-test mean minus Intervention baseline mean) minus (Control post-test mean minus Control baseline mean)] at 6- and 9-months between treatment groups, adjusted for clustering

^2^Adjusted difference is (odds of a higher score at follow-up compared to baseline in Intervention)/(odds of a higher of a higher score at follow-up compared to baseline in Control), adjusted for clustering

**Table 4:** Changes in motivation, self-efficacy, externalizing behaviors, and quality of life

**Secondary outcomes**		**Participants (clusters)**		**Mean change from baseline (SD)**		**Adjusted difference at follow-up**	
	**Time**	**Control**	**Intervention**	**Control**	**Intervention**	**Coefficient (95% CI)**	***P* value**
Motivation for physical activity, units	6 months	124 (14)	130 (14)	−0.05 (0.76)	0.05 (0.70)	1.58 (0.74, 3.38)^2^	0.228
Motivation for physical activity, units	9 months	124 (14)	130 (14)	−0.05 (0.78)	0.08 (0.76)	1.17 (0.55, 2.53)^2^	0.676
HIIT self-efficacy, units	6 months	123 (14)	129 (14)	0.31 (2.19)	0.70 (2.48)	1.70 (0.81, 3.56)^2^	0.150
HIIT self-efficacy, units	9 months	123 (14)	129 (14)	0.17 (2.07)	0.65 (2.19)	2.32 (1.09, 4.95)^2^	**0.030**
Conduct disorders, units	6 months	121 (14)	128 (14)	0.10 (1.39)	0.08 (1.46)	0.89 (0.38, 2.09)^2^	0.784
Conduct disorder, units	9 months	122 (14)	128 (14)	0.04 (1.44)	0.33 (1.57)	1.13 (0.46, 2.75)^2^	0.789
Hyperactivity, units	6 months	121 (14)	129 (14)	0.14 (2.03)	−0.36 (1.87)	0.52 (0.24, 1.09)^2^	0.082
Hyperactivity, units	9 months	122 (14)	129 (14)	−0.02 (2.38)	−0.31 (2.14)	0.57 (0.26, 1.23)^2^	0.148
Quality of life, units	6 months	121 (14)	128 (14)	0.10 (0.16)	0.12 (0.18)	1.06 (0.53, 2.12)^2^	0.868
Quality of life, units	9 months	122 (14)	128 (14)	0.03 (0.19)	0.00 (0.17)	0.98 (0.47, 2.04)^2^	0.961

*HIIT* high-intensity interval training, *SD* standard deviation*, 95% CI* 95% confidence intervals

^1^Adjusted difference [(Intervention post-test mean minus Intervention baseline mean) minus (Control post-test mean minus Control baseline mean)] at 6- and 9-months between treatment groups, adjusted for clustering

^2^Adjusted difference is (odds of a higher score at follow-up compared to baseline in Intervention)/(odds of a higher of a higher score at follow-up compared to baseline in Control), adjusted for clustering

### Process evaluation

Teachers delivered 1.9 ± 1.0 (95% of intended dose) sessions/week in Phases 1–3 (Supplementary Table 10). Researcher observations showed the intervention was delivered with high fidelity (16.0/20 ± 1.8 checklist items; 80%). Average heart rate (65.6% ± 6.6) and peak heart rate (76.2% ± 6.5) during sessions were below prescribed thresholds (i.e., > 80%). Teachers were highly satisfied with the intervention (4.7/5 ± 0.6) and their intention to deliver the program in future was moderate-to-high (4.0/5 ± 0.8). No adverse events were reported.

## Discussion

Our findings suggest special education teachers can be supported to effectively deliver physical activity breaks that improve functional capacity in youth with disability. However, the activity breaks were not of sufficient frequency, duration, or intensity, to achieve clinically important health effects. We also observed positive intervention effects for lower body muscular endurance, resistance training skill competency, HIIT self-efficacy, and hyperactivity among girls. B2La was delivered with a moderate-to-high degree of fidelity and was well received by teachers.

We observed a small improvement in functional capacity that was below our pre-specified MCID threshold, and much smaller than the effect we found in our feasibility study (20.3 m versus 163 m). We attribute this finding to two main factors. First, the original intervention did not explicitly focus on motor competence, and the revised version lowered session intensity to focus on technique. Observations conducted during our feasibility study revealed variability in students’ motor competency, raising concerns about potential injury. In response, we included a preliminary intervention phase focused on improving motor competence and teachers were encouraged to focus on improving students’ technique throughout the intervention. These changes led to a reduction in session intensity, which may explain the smaller intervention effect. Although B2La was originally conceptualized as HIIT, the actual intensity was closer to vigorous-intensity interval training (i.e., 70–75% age-predicted heart rate maximum).

Program drift (i.e., deviation from the original procedures of an intervention during implementation) [41] is an alternate explanation for the ‘voltage drop’ [42] observed. Our protocol asked teachers to deliver two to three sessions/week. Teachers in our feasibility study delivered an average of 2.5 sessions/week, while those in the current study delivered an average of 1.9 sessions/week. It is possible the reduced dosage led to smaller improvements in functional capacity. The effects of scaled-up physical activity interventions are 60% smaller than pre-scale up efficacy trials [43]. A major factor contributing to 'voltage drop' in school-based studies is variability in intervention delivery [42, 44]. Finding ways to support teachers’ implementation of interventions is a priority. As noted by Ryan and colleagues [45], teacher-targeted behavior change techniques are rarely used in school-based teacher-led physical activity interventions. We designed B2La to be scalable and used the Consolidated Framework for Implementation Research [26] to develop a suite of evidence-based strategies to support implementation and sustainment in schools (e.g., professional learning for teachers, action plans, session observations, and executive support).

Schools are ideal settings for physical activity promotion among youth with disability, but previous interventions have lacked generalizability, transferability, and scientific rigor [12]. For example, most school-based interventions targeting this population have been delivered by researchers or paid external providers. Compared with teacher-delivered interventions, those delivered by external providers are typically more effective, but also more expensive and less scalable. We designed B2La to be delivered during scheduled learning support classes by special education teachers, thus representing a new opportunity for youth with disability to be physically active at school [46]. However, compliance with our activity monitoring protocol was low, and the intervention did not increase participants’ physical activity during the school day.

B2La was designed to develop students’ physical literacy. Importantly, our study found the teacher delivered intervention, can improve resistance training skill competence and HIIT self-efficacy in youth with disability. These outcomes may be more important for long-term physical activity behavior than fitness outcomes but are rarely tested in physical activity interventions for youth with disability [47]. As noted by Lai and colleagues [12], there is a need to identify strategies that promote sustainable physical activity among youth with disability. We provided teachers with training, resources, and support to deliver the sessions using the Supportive, Active, Autonomous, Fair and Enjoyable (SAAFE) principles [48]. Guided by self-determination theory [49], the SAAFE principles were designed to promote autonomy supportive teaching and enhance students’ autonomous motivation for physical activity. Although teachers’ delivery adhered to these principles, we did not observe significant changes in participants’ motivation. This is consistent with prior research in youth with disability [50]. These findings further reinforce the importance of providing new physical activity opportunities for young people with disability during the school day. Physical education (PE) represents an important opportunity for young people to be active at school, and there is a large body of research focusing on the inclusion of youth with disability in PE classes [51]. Although many teachers support the idea of inclusion in PE, they often lack the confidence and skills needed to effectively integrate youth with disability in mainstream PE classes [51]. This may explain why students with disabilities experience less motor engagement than their peers without disabilities during mainstream PE lessons [52]. Limited pre-service training and a lack of ongoing professional development in adapted physical education are key barriers, highlighting the importance of targeted training and resources to build teachers’ confidence and competence in supporting students with disabilities [53, 54].

Our null findings for HRQoL align with evidence from a recent review [55], which concluded that physical activity interventions have generally small and inconsistent effects on HRQoL in individuals with disability. Given the multidimensional nature of the HRQoL construct, it is possible that gains achieved through B2La in areas such as physical functioning were overshadowed by adverse changes in other domains. For example, psychological and social wellbeing may have declined due to the broader challenges faced by this population, potentially masking small improvements observed elsewhere. Furthermore, while the short, high-intensity format of B2La was advantageous for implementation, it may not have been optimal for improving HRQoL. Finally, it is plausible that perceived improvements in physical functioning were not fully recognised by students.

B2La did not reduce externalizing problems in the full study sample. Previous reviews of physical activity interventions have reported moderate-to-large improvements in mental health among youth with intellectual disabilities [7] and neurodevelopmental disorders [8, 56]. However, previous physical activity interventions have lacked scalability [12], often involving a small number of participants and a large number of sessions delivered by an external provider over a short period of time. Considering participant variability, we examined the effects in specific sub-groups. Interestingly, we found that girls in the intervention group were significantly less likely to have higher hyperactivity scores at 6-months, compared with girls in the control group. Classroom physical activity breaks have proven efficacy for improving students’ classroom behavior in elementary schools [17], but few studies have tested their effects in secondary schools or among youth with disability [57].

### Strengths and limitations

This appears to be the largest experimental study evaluating the effects of school-based physical activity intervention for youth with disability to date. We designed B2La to be scalable and provided special education teachers with training, resources, and support to deliver the intervention during scheduled lessons. However, there are some limitations that should be noted. First, compliance with our accelerometer monitoring protocol was low and we observed large changes in students’ behavior in both the control and intervention groups, suggesting potential reactivity. Second, we did not include a gold standard measure of body composition. At 9-months we observed a significant group-by-time effect for BMI in favor of the control group (i.e., an increase in BMI among those in the intervention group). Upon further inspection, we found a large increase in BMI among youth with disability with a healthy body weight at baseline and a small non-significant increase among those who were overweight or obese. It is possible that the increase in BMI was due to an increase in muscle mass and/or bone mineral density, both of which respond favorably to resistance training [58]. Finally, we did not include a long-term follow-up assessment to determine if changes in fitness and motor competence were sustained. We conducted our study over one school year because we targeted older adolescents (i.e., > 15 years), many of whom leave school once they turn 16.

### Adaptations and future directions

Consistent with our design, most teachers successfully adapted the intervention to meet the specific needs of their students. For example, students in special support schools required substantial teacher assistance during exercise intervals (e.g., hand support during step-ups), and both these students and those with visual impairments typically participated in a one-to-one teacher–student ratio. While workshop time was allocated for teachers to consider individual student needs, providing additional time for this task in future programs may further enhance implementation. In addition, future interventions should seek to balance improvements in fitness with the development of physical literacy, ensuring that youth with disability gain both the capacity, confidence, and motivation to be active. Importantly, sessions should be enjoyable and designed to prepare students for lifelong participation in physical activity, thereby maximizing the long-term health and wellbeing benefits.

## Conclusions

Our findings highlight the potential health and learning benefits of providing youth with disability opportunities to be physically active during the school day. While the improvements in fitness outcomes were small, they were accompanied by gains in exercise confidence and motor competence, which may be important precursors for continued participation in physical activity beyond school. We also observed reductions in externalizing problems among girls, which may have positive implications for classroom learning and behavior. Although many schools strive to integrate students with disabilities into mainstream PE, this approach often fails to deliver consistent health benefits—particularly in the senior years of schooling (Years 11 and 12), where physical activity is no longer mandated. Our study demonstrates that special education teachers can be trained to deliver brief physical activity breaks, and even modest improvements in functional capacity, muscular fitness, and psychosocial outcomes may provide meaningful benefits for this population.

## Supplementary Information

Supplementary Material 1.

Supplementary Material 2.

## Data Availability

The datasets used in the current study are available from the corresponding author on reasonable request.

## Abbreviations

BMI: Body mass index
B2L: Burn 2 Learn
B2La: Burn 2 Learn adapted
HIIT: High-intensity interval training
HRQoL: Health-related quality of life
MCID: Minimal clinically important difference
PE: Physical education
SAAFE: Supportive, active, autonomous, fair and enjoyable
