# Supplementary material for: Scalable physical activity intervention for youth with disability: Burn 2 Learn adapted cluster randomized controlled trial
Source: Int J Behav Nutr Phys Act. 2025 Oct 9;22:125. doi: 10.1186/s12966-025-01829-1 (PMC12509404; doi:10.1186/s12966-025-01829-1)

**Supplementary Table 1: Baseline, 6- and 9-month means, standard deviations, and ICC values for all outcomes**

| **Outcomes** | **Baseline mean (SD)** | | **6-month mean (SD)** | | **9-month mean (SD)** | |  |
| --- | --- | --- | --- | --- | --- | --- | --- |
|  | **Control** | **Intervention** | **Control** | **Intervention** | **Control** | **Intervention** | **ICC^1^** |
| Functional capacity, m | 484.30 (120.67) | 469.74 (89.79) | 476.27 (116.10) | 480.60 (83.95) | 473.42 (107.93) | 471.09 (113.89) | 0.031 |
| Upper body muscular endurance, reps | 5.85 (7.39) | 4.85 (6.44) | 6.58 (7.30) | 6.61 (7.47) | 6.56 (7.31) | 7.23 (6.98) | 0.104 |
| Lower body muscular endurance, reps | 11.80 (4.26) | 11.19 (2.68) | 11.01 (3.98) | 11.61 (2.62) | 11.75 (3.98) | 12.15 (2.84) | 0.161 |
| Body mass index, kg/m^2^ | 24.12 (6.52) | 24.84 (7.24) | 24.29 (6.43) | 25.12 (7.07) | 24.12 (6.30) | 24.93 (7.54) | <.001 |
| MVPA, mins/weekday | 30.02 (20.87) | 37.92 (19.55) | 37.03 (31.77) | 30.33 (19.51) | 37.00 (19.59) | 35.44 (23.20) | 0.157 |
| MVPA, mins/weekend day | 29.96 (26.25) | 31.45 (22.79) | 37.23 (37.00) | 24.17 (17.74) | 24.10 (19.88) | 16.99 (14.05) | 0.144 |
| Steps per weekday | 8,300 (3,356) | 9,380 (3,364) | 9,616 (4,008) | 9,267 (2,944) | 10,385 (3,348) | 9,084 (3,199) | <.001 |
| Steps per weekend day | 7,888 (3,627) | 7,875 (3,359) | 9,154 (4,350) | 7,592 (3,374) | 7,914 (3,448) | 6,332 (2,944) | <.001 |
| Resistance training skill competence, units | 13.47 (5.03) | 11.42 (4.83) | 15.97 (7.56) | 18.52 (7.18) | 16.59 (7.78) | 18.60 (8.18) | 0.140 |
| Motivation for physical activity, units | 2.74 (0.87) | 2.69 (0.92) | 2.68 (0.86) | 2.75 (0.97) | 2.74 (0.86) | 2.74 (0.91) | 0.117 |
| HIIT self-efficacy, units | 5.68 (2.37) | 5.45 (2.30) | 5.96 (2.28) | 6.35 (2.42) | 5.74 (2.56) | 6.44 (2.16) | 0.134 |
| Conduct disorders, units | 1.33 (1.88) | 1.68 (2.20) | 1.52 (1.87) | 1.76 (2.29) | 1.73 (2.23) | 1.67 (1.83) | 0.190 |
| Hyperactivity, units | 4.26 (2.84) | 4.48 (2.64) | 4.44 (2.88) | 4.18 (2.76) | 4.57 (2.24) | 4.24 (2.52) | 0.147 |
| Quality of life, units | 0.77 (0.21) | 0.75 (0.19) | 0.88 (0.10) | 0.86 (0.11) | 0.78 (0.21) | 0.76 (0.20) | <.001 |

*Note.* HIIT = high-intensity interval training, MVPA = moderate-to-vigorous physical activity, SD = standard deviation, m = meters, reps = repetitions.

^1^ICC=Unconditional intraclass correlation coefficient reflecting similarity of individual observations from the same school

**Supplementary Table 2. Subgroup analysis of fitness, physical activity, and motor competence by intellectual disability**

| **Outcome** |  | **Participants** | | **Interaction** |  |  |
| --- | --- | --- | --- | --- | --- | --- |
|  | **Time** | **Control** | **Intervention** | ***P* value** | **Subgroup** | **Estimate (95% CI)** |
| Functional capacity, m | 6 months | 121 | 123 | 0.604 | No intellectual disability | **27.58 (1.60, 53.55)** ^1^ |
|  |  |  |  |  | Mild intellectual disability | 24.18 (-4.75, 53.11) ^1^ |
|  |  |  |  |  | Moderate to severe intellectual disability | 6.70 (-26.69, 40.10) ^1^ |
| Functional capacity, m | 9 months | 123 | 127 | 0.867 | No intellectual disability | 24.83 (-3.77, 53.42) ^1^ |
|  |  |  |  |  | Mild intellectual disability | 26.45 (-5.38, 58.28) ^1^ |
|  |  |  |  |  | Moderate to severe intellectual disability | 3.79 (-29.77, 37.34) ^1^ |
| Upper body muscular endurance, reps | 6 months | 117 | 115 | 0.163 | No intellectual disability | 1.79 (0.48, 6.68) ^2^ |
|  |  |  |  |  | Mild intellectual disability | 0.53 (0.12, 2.29) ^2^ |
|  |  |  |  |  | Moderate to severe intellectual disability | 5.74 (0.70, 46.92) ^2^ |
| Upper body muscular endurance, reps | 9 months | 120 | 116 | 0.751 | No intellectual disability | 2.19 (0.55, 8.72) ^2^ |
|  |  |  |  |  | Mild intellectual disability | 1.29 (0.31, 5.44) ^2^ |
|  |  |  |  |  | Moderate to severe intellectual disability | 1.96 (0.25, 15.05) ^2^ |
| Lower body muscular endurance, reps | 6 months | 122 | 126 | 0.684 | No intellectual disability | 0.90 (-0.36, 2.17) ^1^ |
|  |  |  |  |  | Mild intellectual disability | 1.35 (-0.10, 2.80) ^1^ |
|  |  |  |  |  | Moderate to severe intellectual disability | 0.38 (-1.27, 2.03) ^1^ |
| Lower body muscular endurance, reps | 9 months | 122 | 127 | 0.894 | No intellectual disability | 0.88 (-0.50, 2.26) ^1^ |
|  |  |  |  |  | Mild intellectual disability | 1.42 (-0.15, 2.99) ^1^ |
|  |  |  |  |  | Moderate to severe intellectual disability | 0.55 (-1.11, 2.22) ^1^ |
| Body mass index, kg/m^2^ | 6 months | 122 | 128 | 0.421 | No intellectual disability | -0.18 (-0.73, 0.36) ^1^ |
|  |  |  |  |  | Mild intellectual disability | 0.25 (-0.37, 0.87) ^1^ |
|  |  |  |  |  | Moderate to severe intellectual disability | 0.35 (-0.38, 1.08) ^1^ |
| Body mass index, kg/m^2^ | 9 months | 123 | 128 | 0.457 | No intellectual disability | 0.32 (-0.75, 1.39) ^1^ |
|  |  |  |  |  | Mild intellectual disability | **1.63 (0.46, 2.80)** ^1^ |
|  |  |  |  |  | Moderate to severe intellectual disability | 0.57 (-0.67, 1.82) ^1^ |
| MVPA, mins/weekday | 6 months | 74 | 83 | 0.216 | No intellectual disability | 0.09 (0.01, 0.76) ^2^ |
|  |  |  |  |  | Mild intellectual disability | 0.41 (0.05, 3.60) ^2^ |
|  |  |  |  |  | Moderate to severe intellectual disability | 1.52 (0.11, 20.59) ^2^ |
| MVPA, mins/weekday | 9 months | 74 | 85 | 0.363 | No intellectual disability | 0.30 (0.03, 2.88) ^2^ |
|  |  |  |  |  | Mild intellectual disability | 0.49 (0.04, 6.24) ^2^ |
|  |  |  |  |  | Moderate to severe intellectual disability | 0.18 (0.01, 4.68) ^2^ |
| MVPA, mins/weekend day | 6 months | 52 | 62 | 0.412 | No intellectual disability | 0.12 (0.00, 7.25) ^2^ |
|  |  |  |  |  | Mild intellectual disability | 1.79 (0.10, 32.66) ^2^ |
|  |  |  |  |  | Moderate to severe intellectual disability | 1.66 (0.05, 52.77) ^2^ |
| MVPA, mins/weekend day | 9 months | 59 | 66 | 0.412 | No intellectual disability | 0.13 (0.01, 1.95) ^2^ |
|  |  |  |  |  | Mild intellectual disability | 0.74 (0.04, 12.18) ^2^ |
|  |  |  |  |  | Moderate to severe intellectual disability | 2.26 (0.03, 163.55) ^2^ |
| Steps/weekday | 6 months | 80 | 80 | 0.362 | No intellectual disability | -2180.75 (-4381.51, 20.02) ^1^ |
|  |  |  |  |  | Mild intellectual disability | -522.20 (-2812.35, 1767.95) ^1^ |
|  |  |  |  |  | Moderate to severe intellectual disability | 87 (-2508, 2682) ^1^ |
| Steps/weekday | 9 months | 80 | 82 | 0.616 | No intellectual disability | -1958 (-4425, 510) ^1^ |
|  |  |  |  |  | Mild intellectual disability | -418 (-3412, 2575) ^1^ |
|  |  |  |  |  | Moderate to severe intellectual disability | -2400 (-6297, 1498) ^1^ |
| Steps/weekend day | 6 months | 54 | 63 | 0.468 | No intellectual disability | 0.13 (0.00, 4.57) ^2^ |
|  |  |  |  |  | Mild intellectual disability | 0.94 (0.06, 15.62) ^2^ |
|  |  |  |  |  | Moderate to severe intellectual disability | 1.48 (0.06, 39.72) ^2^ |
| Steps/weekend day | 9 months | 60 | 66 | 0.283 | No intellectual disability | 0.09 (0.01, 1.06) ^2^ |
|  |  |  |  |  | Mild intellectual disability | 0.94 (0.06, 14.36) ^2^ |
|  |  |  |  |  | Moderate to severe intellectual disability | 3.11 (0.06, 156.80) ^2^ |
| Resistance training skill competence | 6 months | 118 | 119 | 0.599 | No intellectual disability | **15.24 (4.02, 57.77)** ^2^ |
|  |  |  |  |  | Mild intellectual disability | **7.18 (1.71, 30.15)** ^2^ |
|  |  |  |  |  | Moderate to severe intellectual disability | **20.23 (3.01, 135.87)** ^2^ |
| Resistance training skill competence | 9 months | 119 | 119 | 0.891 | No intellectual disability | **13.88 (3.38, 56.94)** ^2^ |
|  |  |  |  |  | Mild intellectual disability | **10.35 (2.35, 45.55)** ^2^ |
|  |  |  |  |  | Moderate to severe intellectual disability | **13.64 (2.27, 81.80)** ^2^ |

^1^Adjusted difference is the difference in change from baseline in treatment 2 compared to treatment 1 [(Intervention follow-up minus Intervention baseline) - (Control follow-up minus Control baseline)], with the moderator held constant, adjusted for clustering.
^2^Adjusted difference is (odds of a higher score at follow up compared to baseline in Intervention)/(odds of a higher of a higher score at follow-up compared to baseline in Control), with the moderator held constant, adjusted for clustering.  
*Note.* HIIT = high-intensity interval training, 95% CI = 95% confidence intervals**.**

**Supplementary Table 3. Subgroup analysis of motivation, self-efficacy, externalizing behaviors, and quality of life by intellectual disability**

| **Outcome** |  | **Participants** | | **Interaction** |  |  |
| --- | --- | --- | --- | --- | --- | --- |
|  | **Time** | **Control** | **Intervention** | ***P* value** | **Subgroup** | **Estimate**^1^ **(95% CI)** |
| Motivation for physical activity | 6 months | 124 | 130 | 0.751 | No intellectual disability | 1.41 (0.45, 4.41) |
|  |  |  |  |  | Mild intellectual disability | 2.49 (0.68, 9.17) |
|  |  |  |  |  | Moderate to severe intellectual disability | 1.31 (0.26, 6.53) |
| Motivation for physical activity | 9 months | 124 | 130 | 0.961 | No intellectual disability | 1.05 (0.32, 3.37) |
|  |  |  |  |  | Mild intellectual disability | 1.20 (0.31, 4.58) |
|  |  |  |  |  | Moderate to severe intellectual disability | 1.36 (0.27, 6.82) |
| HIIT self-efficacy | 6 months | 123 | 129 | 0.486 | No intellectual disability | 1.22 (0.41, 3.59) |
|  |  |  |  |  | Mild intellectual disability | 1.60 (0.46, 5.63) |
|  |  |  |  |  | Moderate to severe intellectual disability | 3.33 (0.62, 17.74) |
| HIIT self-efficacy | 9 months | 123 | 129 | 0.486 | No intellectual disability | 2.10 (0.66, 6.72) |
|  |  |  |  |  | Mild intellectual disability | 1.44 (0.39, 5.30) |
|  |  |  |  |  | Moderate to severe intellectual disability | **7.39 (1.50, 36.30)** |
| Conduct disorders | 6 months | 121 | 128 | 0.343 | No intellectual disability | 0.78 (0.21, 2.89) |
|  |  |  |  |  | Mild intellectual disability | 0.32 (0.07, 1.51) |
|  |  |  |  |  | Moderate to severe intellectual disability | 3.03 (0.59, 15.52) |
| Conduct disorders | 9 months | 121 | 128 | 0.343 | No intellectual disability | 1.06 (0.26, 4.32) |
|  |  |  |  |  | Mild intellectual disability | 0.86 (0.17, 4.24) |
|  |  |  |  |  | Moderate to severe intellectual disability | 1.29 (0.23, 7.26) |
| Hyperactivity | 6 months | 121 | 129 | 0.564 | No intellectual disability | 0.21 (0.05, 0.81) |
|  |  |  |  |  | Mild intellectual disability | 0.21 (0.05, 0.81) |
|  |  |  |  |  | Moderate to severe intellectual disability | 0.82 (0.20, 3.39) |
| Hyperactivity | 9 months | 122 | 129 | 0.564 | No intellectual disability | 0.41 (0.12, 1.36) |
|  |  |  |  |  | Mild intellectual disability | 0.40 (0.10, 1.61) |
|  |  |  |  |  | Moderate to severe intellectual disability | 1.07 (0.25, 4.53) |
| Quality of life | 6 months | 121 | 128 | 0.383 | No intellectual disability | 1.01 (0.36, 2.82) |
|  |  |  |  |  | Mild intellectual disability | 1.68 (0.52, 5.49) |
|  |  |  |  |  | Moderate to severe intellectual disability | 0.64 (0.14, 2.92) |
| Quality of life | 9 months | 122 | 128 | 0.383 | No intellectual disability | 1.79 (0.58, 5.52) |
|  |  |  |  |  | Mild intellectual disability | 0.73 (0.21, 2.59) |
|  |  |  |  |  | Moderate to severe intellectual disability | 0.39 (0.08, 1.88) |

^1^Adjusted difference is (odds of a higher score at follow up compared to baseline in Intervention)/(odds of a higher of a higher score at follow-up compared to baseline in Control), with the moderator held constant, adjusted for clustering.  
*Note.* HIIT = high-intensity interval training, 95% CI = 95% confidence intervals**.**

**Supplementary Table 4. Subgroup analysis of fitness, physical activity and motor competence by socioeconomic status**

| **Outcome** |  | **Participants** | | **Interaction** |  |  |
| --- | --- | --- | --- | --- | --- | --- |
|  | **Time** | **Control** | **Intervention** | ***P* value** | **Subgroup** | **Estimate (95% CI)** |
| Functional capacity, m | 6 months | 119 | 116 | 0.331 | Lowest tertile | 51.11 (-18.69, 120.91) ^1^ |
|  |  |  |  |  | Middle tertile | 19.81 (-1.82, 41.43) ^1^ |
|  |  |  |  |  | Highest tertile | 0.03 (-29.61, 29.67) ^1^ |
| Functional capacity, m | 9 months | 119 | 117 | 0.172 | Lowest tertile | 9.25 (-55.85, 74.35) ^1^ |
|  |  |  |  |  | Middle tertile | **31.70 (8.41, 54.98)** ^1^ |
|  |  |  |  |  | Highest tertile | -11.53 (-44.55, 21.49) ^1^ |
| Upper body muscular endurance, reps | 6 months | 113 | 106 | 0.959 | Lowest tertile | 2.34 (0.12, 44.83) ^2^ |
|  |  |  |  |  | Middle tertile | 1.55 (0.51, 4.72) ^2^ |
|  |  |  |  |  | Highest tertile | 1.56 (0.28, 8.74) ^2^ |
| Upper body muscular endurance, reps | 9 months | 116 | 107 | 0.612 | Lowest tertile | 1.05 (0.06, 19.11) ^2^ |
|  |  |  |  |  | Middle tertile | 3.20 (0.94, 10.86) ^2^ |
|  |  |  |  |  | Highest tertile | 0.75 (0.12, 4.61) ^2^ |
| Lower body muscular endurance, reps | 6 months | 118 | 116 | 0.688 | Lowest tertile | 2.08 (-1.10, 5.26) ^1^ |
|  |  |  |  |  | Middle tertile | 0.63 (-0.50, 1.75) ^1^ |
|  |  |  |  |  | Highest tertile | 0.94 (-0.55, 2.44) ^1^ |
| Lower body muscular endurance, reps | 9 months | 118 | 117 | 0.277 | Lowest tertile | **4.13 (0.63, 7.64)** ^1^ |
|  |  |  |  |  | Middle tertile | 1.09 (-0.09, 2.27) ^1^ |
|  |  |  |  |  | Highest tertile | 0.08 (-1.57, 1.72) ^1^ |
| Body mass index, kg/m^2^ | 6 months | 118 | 118 | 0.181 | Lowest tertile | 0.14 (-1.16, 1.44) ^1^ |
|  |  |  |  |  | Middle tertile | -0.14 (-0.62, 0.34) ^1^ |
|  |  |  |  |  | Highest tertile | 0.62 (-0.03, 1.27) ^1^ |
| Body mass index, kg/m^2^ | 9 months | 119 | 118 | 0.485 | Lowest tertile | 1.86 (-0.53, 4.25) ^1^ |
|  |  |  |  |  | Middle tertile | 0.24 (-0.64, 1.12) ^1^ |
|  |  |  |  |  | Highest tertile | 0.91 (-0.30, 2.12) ^1^ |
| MVPA, mins/weekday | 6 months | 71 | 78 | 0.728 | Lowest tertile | 0.47 (0.00, 48.51) ^2^ |
|  |  |  |  |  | Middle tertile | 0.86 (0.09, 8.07) ^2^ |
|  |  |  |  |  | Highest tertile | 0.71 (0.05, 11.18) ^2^ |
| MVPA, mins/weekday | 9 months | 71 | 80 | 0.861 | Lowest tertile | 0.28 (0.00, 160.65) ^2^ |
|  |  |  |  |  | Middle tertile | 0.51 (0.06, 4.74) ^2^ |
|  |  |  |  |  | Highest tertile | 1.61 (0.00, 635.41) ^2^ |
| MVPA, mins/weekend day | 6 months | 50 | 58 | 0.974 | Lowest tertile | 2.34 (0.12, 44.83) ^2^ |
|  |  |  |  |  | Middle tertile | 1.55 (0.51, 4.72) ^2^ |
|  |  |  |  |  | Highest tertile | 1.56 (0.28, 8.74) ^2^ |
| MVPA, mins/weekend day | 9 months | 57 | 61 | **0.088** | Lowest tertile | 0.00 (0.00, 0.32) ^2^ |
|  |  |  |  |  | Middle tertile | 2.54 (0.39, 16.62) ^2^ |
|  |  |  |  |  | Highest tertile | 0.16 (0.00, 4.95) ^2^ |
| Steps/weekday | 6 months | 77 | 75 | 0.315 | Lowest tertile | -1172 (-5539, 3194) ^1^ |
|  |  |  |  |  | Middle tertile | -1509 (-3381, 362) ^1^ |
|  |  |  |  |  | Highest tertile | 866 (-1672, 3404) ^1^ |
| Steps/weekday | 9 months | 77 | 77 | 0.560 | Lowest tertile | -3220 (-9277, 2836) ^1^ |
|  |  |  |  |  | Middle tertile | -1732 (-3867, 402) ^1^ |
|  |  |  |  |  | Highest tertile | 1561 (-4386, 7509) ^1^ |
| Steps/weekend day | 6 months | 52 | 59 | 0.853 | Lowest tertile | 1.85 (0.03, 101.92) ^2^ |
|  |  |  |  |  | Middle tertile | 0.53 (0.09, 3.24) ^2^ |
|  |  |  |  |  | Highest tertile | 0.76 (0.09, 6.46) ^2^ |
| Steps/weekend day | 9 months | 58 | 61 | 0.559 | Lowest tertile | 0.04 (0.00, 2.42) ^2^ |
|  |  |  |  |  | Middle tertile | 0.93 (0.15, 5.80) ^2^ |
|  |  |  |  |  | Highest tertile | 1.34 (0.05, 33.03) ^2^ |
| Resistance training skill competence | 6 months | 114 | 110 | 0.971 | Lowest tertile | 2.91 (0.12, 70.73) ^2^ |
|  |  |  |  |  | Middle tertile | **4.21 (1.45, 12.20)** ^2^ |
|  |  |  |  |  | Highest tertile | 3.91 (0.86, 17.72) ^2^ |
| Resistance training skill competence | 9 months | 115 | 110 | 0.996 | Lowest tertile | 1.94 (0.07, 52.18) ^2^ |
|  |  |  |  |  | Middle tertile | **3.22 (1.08, 9.55)** ^2^ |
|  |  |  |  |  | Highest tertile | 3.66 (0.79, 17.01) ^2^ |

^1^Adjusted difference is the difference in change from baseline in treatment 2 compared to treatment 1 [(Intervention follow-up minus Intervention baseline) - (Control follow-up minus Control baseline)], with the moderator held constant, adjusted for clustering.
^2^Adjusted difference is (odds of a higher score at follow up compared to baseline in Intervention)/(odds of a higher of a higher score at follow-up compared to baseline in Control), with the moderator held constant, adjusted for clustering.  
*Note.* HIIT = high-intensity interval training. 95% CI = 95% confidence intervals**.**

**Supplementary Table 5. Subgroup analysis of motivation, self-efficacy, externalizing behaviors, and quality of life by socioeconomic status**

| **Outcome** |  | **Participants** | | **Interaction** |  |  |
| --- | --- | --- | --- | --- | --- | --- |
|  | **Time** | **Control** | **Intervention** | ***P* value** | **Subgroup** | **Estimate**^1^ **(95% CI)** |
| Motivation for physical activity | 6 months | 120 | 119 | 0.567 | Lowest tertile | 6.28 (0.37, 106.62) |
|  |  |  |  |  | Middle tertile | 1.62 (0.59, 4.48) |
|  |  |  |  |  | Highest tertile | 1.23 (0.27, 5.49) |
| Motivation for physical activity | 9 months | 120 | 119 | 0.731 | Lowest tertile | 0.78 (0.04, 14.39) |
|  |  |  |  |  | Middle tertile | 1.60 (0.57, 4.45) |
|  |  |  |  |  | Highest tertile | 0.66 (0.15, 2.97) |
| HIIT self-efficacy | 6 months | 119 | 119 | **0.067** | Lowest tertile | 1.93 (0.13, 27.78) |
|  |  |  |  |  | Middle tertile | 0.75 (0.27, 2.05) |
|  |  |  |  |  | Highest tertile | **6.54 (1.46, 29.27)** |
| HIIT self-efficacy | 9 months | 119 | 119 | **0.067** | Lowest tertile | 0.80 (0.04, 14.67) |
|  |  |  |  |  | Middle tertile | 1.99 (0.72, 5.50) |
|  |  |  |  |  | Highest tertile | 2.62 (0.58, 11.76) |
| Conduct disorders | 6 months | 118 | 117 | 0.579 | Lowest tertile | 1.21 (0.04, 35.93) |
|  |  |  |  |  | Middle tertile | 0.81 (0.25, 2.69) |
|  |  |  |  |  | Highest tertile | 2.26 (0.42, 11.99) |
| Conduct disorders | 9 months | 119 | 117 | 0.741 | Lowest tertile | 4.16 (0.16, 109.46) |
|  |  |  |  |  | Middle tertile | 0.87 (0.25, 3.03) |
|  |  |  |  |  | Highest tertile | 2.08 (0.36, 12.12) |
| Hyperactivity | 6 months | 118 | 118 | **0.081** | Lowest tertile | 28.93 (0.86, 970.68) |
|  |  |  |  |  | Middle tertile | 0.53 (0.18, 1.53) |
|  |  |  |  |  | Highest tertile | 0.40 (0.10, 1.56) |
| Hyperactivity | 9 months | 119 | 118 | 0.165 | Lowest tertile | 0.79 (0.04, 15.28) |
|  |  |  |  |  | Middle tertile | 0.57 (0.20, 1.69) |
|  |  |  |  |  | Highest tertile | 0.54 (0.13, 2.18) |
| Quality of life | 6 months | 117 | 119 | 0.483 | Lowest tertile | 0.27 (0.02, 4.32) |
|  |  |  |  |  | Middle tertile | 1.15 (0.44, 3.02) |
|  |  |  |  |  | Highest tertile | 1.59 (0.43, 5.79) |
| Quality of life | 9 months | 118 | 119 | 0.545 | Lowest tertile | 1.57 (0.11, 21.34) |
|  |  |  |  |  | Middle tertile | 0.89 (0.33, 2.38) |
|  |  |  |  |  | Highest tertile | 2.39 (0.57, 10.06) |

^1^Adjusted difference is (odds of a higher score at follow up compared to baseline in Intervention)/(odds of a higher of a higher score at follow-up compared to baseline in Control), with the moderator held constant, adjusted for clustering.
*Note.* HIIT = high-intensity interval training, 95% CI = 95% confidence intervals.

**Supplementary Table 6. Subgroup analysis of fitness, physical activity, and motor competence by sex**

| **Outcome** |  | **Participants** | | **Interaction** |  |  |
| --- | --- | --- | --- | --- | --- | --- |
|  | **Time** | **Control** | **Intervention** | ***P* value** | **Subgroup** | **Estimate (95% CI)** |
| Functional capacity, m | 6 months | 123 | 126 | 0.536 | Female | 29.05 (-4.31, 62.42) ^1^ |
|  |  |  |  |  | Male | 16.89 (-2.81, 36.58) ^1^ |
| Functional capacity, m | 9 months | 123 | 127 | 0.752 | Female | 18.75 (-17.18, 54.67) ^1^ |
|  |  |  |  |  | Male | 16.49 (-4.41, 37.40) ^1^ |
| Upper body muscular endurance, reps | 6 months | 117 | 115 | 0.145 | Female | 0.39 (0.06, 2.71) ^2^ |
|  |  |  |  |  | Male | 1.91 (0.66, 5.56) ^2^ |
| Upper body muscular endurance, reps | 9 months | 120 | 116 | 0.309 | Female | 2.14 (0.28, 16.41) ^2^ |
|  |  |  |  |  | Male | 1.51 (0.51, 4.48) ^2^ |
| Lower body muscular endurance, reps | 6 months | 122 | 126 | 0.211 | Female | -0.13 (-1.78, 1.52) ^1^ |
|  |  |  |  |  | Male | **1.09 (0.12, 2.06)** ^1^ |
| Lower body muscular endurance, reps | 9 months | 122 | 127 | 0.422 | Female | 0.61 (-1.16, 2.38) ^1^ |
|  |  |  |  |  | Male | **1.02 (0.00, 2.05)** ^1^ |
| Body mass index, kg/m^2^ | 6 months | 122 | 128 | 0.944 | Female | 0.12 (-0.58, 0.82) ^1^ |
|  |  |  |  |  | Male | 0.09 (-0.33, 0.52) ^1^ |
| Body mass index, kg/m^2^ | 9 months | 123 | 128 | 0.402 | Female | 0.20 (-1.16, 1.55) ^1^ |
|  |  |  |  |  | Male | **1.17 (0.40, 1.94)** ^1^ |
| MVPA, mins/weekday | 6 months | 74 | 83 | 0.162 | Female | 0.06 (0.00, 1.79) ^2^ |
|  |  |  |  |  | Male | 0.72 (0.13, 4.10) ^2^ |
| MVPA, mins/weekday | 9 months | 74 | 85 | 0.214 | Female | 0.36 (0.01, 10.34) ^2^ |
|  |  |  |  |  | Male | 0.68 (0.11, 4.20) ^2^ |
| MVPA, mins/weekend day | 6 months | 52 | 62 | 0.204 | Female | 0.05 (0.00, 10.23) ^2^ |
|  |  |  |  |  | Male | 1.26 (0.10, 16.40) ^2^ |
| MVPA, mins/weekend day | 9 months | 59 | 66 | **0.084** | Female | 11.26 (0.02, 5271.28) ^2^ |
|  |  |  |  |  | Male | 0.24 (0.02, 3.28) ^2^ |
| Steps/weekday | 6 months | 80 | 80 | **0.091** | Female | **-3456 (-6661, -252)** ^1^ |
|  |  |  |  |  | Male | -373 (-1974, 1228) ^1^ |
| Steps/weekday | 9 months | 80 | 82 | 0.422 | Female | -2806 (-6521, 909) ^1^ |
|  |  |  |  |  | Male | -947 (-2897, 1003) ^1^ |
| Steps/weekend day | 6 months | 54 | 63 | 0.815 | Female | 0.52 (0.01, 51.99) ^2^ |
|  |  |  |  |  | Male | 0.33 (0.03, 3.25) ^2^ |
| Steps/weekend day | 9 months | 60 | 66 | 0.278 | Female | 5.72 (0.06, 520.15) ^2^ |
|  |  |  |  |  | Male | 0.21 (0.03, 1.66) ^2^ |
| Resistance training skill competency | 6 months | 118 | 119 | 0.683 | Female | **20.45 (2.81, 149.10)** ^2^ |
|  |  |  |  |  | Male | **13.44 (4.60, 39.22)** ^2^ |
| Resistance training skill competency | 9 months | 119 | 119 | 0.482 | Female | **38.01 (5.30, 272.90)** ^2^ |
|  |  |  |  |  | Male | **10.45 (3.69, 29.62)** ^2^ |

^1^Adjusted difference is the difference in change from baseline in treatment 2 compared to treatment 1 [(Intervention follow-up minus Intervention baseline) - (Control follow-up minus Control baseline)], with the moderator held constant, adjusted for clustering.
^2^Adjusted difference is (odds of a higher score at follow up compared to baseline in Intervention)/(odds of a higher of a higher score at follow-up compared to baseline in Control), with the moderator held constant, adjusted for clustering.
*Note.* HIIT = high-intensity interval training, 95% CI = 95% confidence intervals**.**

**Supplementary Table 7. Subgroup analysis of motivation, self-efficacy, externalizing behaviors, and quality of life by sex**

| **Outcome** |  | **Participants** | | **Interaction** |  |  |
| --- | --- | --- | --- | --- | --- | --- |
|  | **Time** | **Control** | **Intervention** | ***P* value** | **Subgroup** | **Estimate**^1^ **(95% CI)** |
| Motivation for physical activity | 6 months | 124 | 130 | 0.712 | Female | 1.31 (0.27, 6.28) |
|  |  |  |  |  | Male | 1.81 (0.72, 4.53) |
| Motivation for physical activity | 9 months | 124 | 130 | 0.980 | Female | 1.17 (0.22, 6.11) |
|  |  |  |  |  | Male | 1.06 (0.43, 2.62) |
| HIIT self-efficacy | 6 months | 123 | 129 | 0.789 | Female | 2.14 (0.47, 9.71) |
|  |  |  |  |  | Male | 1.71 (0.70, 4.13) |
| HIIT self-efficacy | 9 months | 123 | 129 | 0.895 | Female | 3.45 (0.73, 16.26) |
|  |  |  |  |  | Male | 2.30 (0.94, 5.60) |
| Conduct disorders | 6 months | 121 | 128 | 0.241 | Female | 0.36 (0.07, 1.93) |
|  |  |  |  |  | Male | 1.14 (0.40, 3.24) |
| Conduct disorders | 9 months | 122 | 128 | 0.333 | Female | 0.99 (0.16, 6.04) |
|  |  |  |  |  | Male | 1.08 (0.38, 3.10) |
| Hyperactivity | 6 months | 121 | 129 | 0.230 | Female | **0.21 (0.05, 0.95)** |
|  |  |  |  |  | Male | 0.60 (0.24, 1.49) |
| Hyperactivity | 9 months | 122 | 129 | 0.435 | Female | 0.33 (0.07, 1.48) |
|  |  |  |  |  | Male | 0.62 (0.25, 1.57) |
| Quality of life | 6 months | 121 | 128 | 0.805 | Female | 1.28 (0.30, 5.37) |
|  |  |  |  |  | Male | 1.05 (0.45, 2.44) |
| Quality of life | 9 months | 122 | 128 | 0.956 | Female | 1.20 (0.27, 5.35) |
|  |  |  |  |  | Male | 0.95 (0.40, 2.27) |

^1^Adjusted difference is (odds of a higher score at follow up compared to baseline in Intervention)/(odds of a higher of a higher score at follow-up compared to baseline in Control), with the moderator held constant, adjusted for clustering.
*Note.* HIIT = high-intensity interval training, 95% CI = 95% confidence intervals**.**

**Supplementary Table 8. Subgroup analysis of fitness, physical activity, and motor competence by weight status**

| **Outcome** |  | **Participants** | | **Interaction** |  |  |
| --- | --- | --- | --- | --- | --- | --- |
|  | **Time** | **Control** | **Intervention** | ***P* value** | **Subgroup** | **Estimate (95% CI)** |
| Functional capacity, m | 6 months | 120 | 125 | 0.821 | Overweight or obese | 17.28 (-8.36, 42.92) ^1^ |
|  |  |  |  |  | Underweight or healthy weight | 21.20 (-1.29, 43.70) ^1^ |
| Functional capacity, m | 9 months | 120 | 125 | **0.037** | Overweight or obese | -5.91 (-33.65, 21.83) ^1^ |
|  |  |  |  |  | Underweight or healthy weight | **38.11 (15.27, 60.96)** ^1^ |
| Upper body muscular endurance, reps | 6 months | 116 | 114 | 0.286 | Overweight or obese | 2.45 (0.59, 10.16) ^2^ |
|  |  |  |  |  | Underweight or healthy weight | 0.93 (0.29, 2.96) ^2^ |
| Upper body muscular endurance, reps | 9 months | 118 | 115 | 0.425 | Overweight or obese | 3.23 (0.70, 14.86) ^2^ |
|  |  |  |  |  | Underweight or healthy weight | 1.11 (0.34, 3.59) ^2^ |
| Lower body muscular endurance, reps | 6 months | 120 | 125 | 0.480 | Overweight or obese | 0.40 (-0.90, 1.71) ^1^ |
|  |  |  |  |  | Underweight or healthy weight | 1.01 (-0.07, 2.09) ^1^ |
| Lower body muscular endurance, reps | 9 months | 120 | 126 | 0.175 | Overweight or obese | -0.06 (-1.45, 1.33) ^1^ |
|  |  |  |  |  | Underweight or healthy weight | **1.64 (0.52, 2.77)** ^1^ |
| Body mass index, kg/m^2^ | 6 months | 121 | 127 | 0.545 | Overweight or obese | -0.02 (-0.16, 0.13) ^1^ |
|  |  |  |  |  | Underweight or healthy weight | 0.04 (-0.08, 0.16) ^1^ |
| Body mass index, kg/m^2^ | 9 months | 121 | 127 | 0.688 | Overweight or obese | 0.53 (-0.52, 1.59) ^1^ |
|  |  |  |  |  | Underweight or healthy weight | **1.12 (0.29, 1.96)** ^1^ |
| MVPA, mins/weekday | 6 months | 74 | 82 | 0.604 | Overweight or obese | **0.25 (0.03, 2.15)** ^2^ |
|  |  |  |  |  | Underweight or healthy weight | 0.49 (0.10, 2.45) ^2^ |
| MVPA, mins/weekday | 9 months | 74 | 84 | 0.287 | Overweight or obese | 1.95 (0.12, 31.62) ^2^ |
|  |  |  |  |  | Underweight or healthy weight | 0.34 (0.06, 1.94) ^2^ |
| MVPA, mins/weekend day | 6 months | 52 | 62 | 0.654 | Overweight or obese | 0.63 (0.02, 17.42) ^2^ |
|  |  |  |  |  | Underweight or healthy weight | 1.38 (0.14, 13.90) ^2^ |
| MVPA, mins/weekend day | 9 months | 59 | 65 | 0.781 | Overweight or obese | 0.17 (0.00, 6.29) ^2^ |
|  |  |  |  |  | Underweight or healthy weight | 0.66 (0.06, 6.96) ^2^ |
| Steps/weekday | 6 months | 78 | 79 | 0.524 | Overweight or obese | -175 (-2395, 2045) ^1^ |
|  |  |  |  |  | Underweight or healthy weight | -1064 (-2770, 643) ^1^ |
| Steps/weekday | 9 months | 78 | 81 | 0.275 | Overweight or obese | 909 (-2326, 4145) ^1^ |
|  |  |  |  |  | Underweight or healthy weight | **-2182 (-4254, -113)** ^1^ |
| Steps/weekend day | 6 months | 54 | 62 | 0.136 | Overweight or obese | 0.15 (0.01, 2.59) ^2^ |
|  |  |  |  |  | Underweight or healthy weight | 1.72 (0.26, 11.24) ^2^ |
| Steps/weekend day | 9 months | 60 | 65 | 0.246 | Overweight or obese | 0.19 (0.01, 3.70) ^2^ |
|  |  |  |  |  | Underweight or healthy weight | 0.80 (0.11, 5.90) ^2^ |
| Resistance training skill competency | 6 months | 117 | 118 | 0.762 | Overweight or obese | **18.03 (4.49, 72.36)** ^2^ |
|  |  |  |  |  | Underweight or healthy weight | **13.94 (4.26, 45.57)** ^2^ |
| Resistance training skill competency | 9 months | 118 | 118 | 0.926 | Overweight or obese | **19.71 (4.63, 83.86)** ^2^ |
|  |  |  |  |  | Underweight or healthy weight | **14.39 (4.52, 45.82)** ^2^ |

^1^Adjusted difference is the difference in change from baseline in treatment 2 compared to treatment 1 [(Intervention follow-up minus Intervention baseline) - (Control follow-up minus Control baseline)], with the moderator held constant, adjusted for clustering.
^2^Adjusted difference is (odds of a higher score at follow up compared to baseline in Intervention)/(odds of a higher of a higher score at follow-up compared to baseline in Control), with the moderator held constant, adjusted for clustering.
*Note.* HIIT = high-intensity interval training, 95% CI = 95% confidence intervals.

**Supplementary Table 9. Subgroup analysis of motivation, self-efficacy, externalizing behaviors, and quality of life by weight status**

| **Outcome** |  | **Participants** | | **Interaction** |  |  |
| --- | --- | --- | --- | --- | --- | --- |
|  | **Time** | **Control** | **Intervention** | ***P* value** | **Subgroup** | **Estimate**^1^ **(95% CI)** |
| Motivation for physical activity | 6 months | 121 | 127 | 0.252 | Overweight or obese | 2.69 (0.83, 8.74) |
|  |  |  |  |  | Underweight or healthy weight | 1.11 (0.40, 3.08) |
| Motivation for physical activity | 9 months | 121 | 127 | 0.439 | Overweight or obese | 1.43 (0.43, 4.77) |
|  |  |  |  |  | Underweight or healthy weight | 1.09 (0.39, 3.02) |
| HIIT self-efficacy | 6 months | 120 | 126 | 0.865 | Overweight or obese | 1.93 (0.60, 6.20) |
|  |  |  |  |  | Underweight or healthy weight | 1.70 (0.65, 4.51) |
| HIIT self-efficacy | 9 months | 120 | 126 | 0.772 | Overweight or obese | 3.04 (0.92, 10.10) |
|  |  |  |  |  | Underweight or healthy weight | 1.86 (0.69, 5.04) |
| Conduct disorders | 6 months | 117 | 125 | 0.969 | Overweight or obese | 0.85 (0.21, 3.46) |
|  |  |  |  |  | Underweight or healthy weight | 0.82 (0.27, 2.45) |
| Conduct disorders | 9 months | 118 | 125 | 0.587 | Overweight or obese | 0.59 (0.12, 2.85) |
|  |  |  |  |  | Underweight or healthy weight | 1.37 (0.45, 4.18) |
| Hyperactivity | 6 months | 117 | 126 | 0.646 | Overweight or obese | 0.42 (0.13, 1.35) |
|  |  |  |  |  | Underweight or healthy weight | 0.59 (0.22, 1.58) |
| Hyperactivity | 9 months | 118 | 126 | **0.092** | Overweight or obese | **0.17 (0.05, 0.61)** |
|  |  |  |  |  | Underweight or healthy weight | 0.95 (0.35, 2.56) |
| Quality of life | 6 months | 119 | 126 | 0.422 | Overweight or obese | 1.52 (0.52, 4.46) |
|  |  |  |  |  | Underweight or healthy weight | 0.87 (0.34, 2.18) |
| Quality of life | 9 months | 119 | 126 | 0.557 | Overweight or obese | 1.43 (0.46, 4.47) |
|  |  |  |  |  | Underweight or healthy weight | 0.67 (0.25, 1.75) |

^1^Adjusted difference is (odds of a higher score at follow up compared to baseline in Intervention)/(odds of a higher of a higher score at follow-up compared to baseline in Control), with the moderator held constant, adjusted for clustering.
*Note.* HIIT = high-intensity interval training, 95% CI = 95% confidence intervals.

**Supplementary Table 10.**  **Process evaluation**

| **Intervention dose (teacher reported)**^1^  Total number of B2La sessions delivered in Phase 1, mean (SD)  Total number of B2La sessions delivered in Phases 2 and 3, mean (SD)  B2La sessions/week in Phases 1 to 3, mean (SD) | 8.7 (6.7)  19.5 (9.1)  1.9 (1.0) |
| --- | --- |
| **Intervention fidelity (session observations)^2^**  *Supportive:* Teacher was supportive and promoted positive student interactions, mean (SD)^3^  *Active:* Session was highly active, and transition time was minimal, mean (SD)^3^  *Autonomous:* Session involved opportunities for student choice, mean (SD)^3^  *Fair:* All students provided with opportunities to experience success, mean (SD)^3^  *Enjoyable:* Session was enjoyable and included a variety of activities, mean (SD)^3^  Overall adherence to SAAFE principles, mean (SD) | 3.4 (0.4)  3.1 (0.6)  2.8 (0.5)  3.3 (0.6)  3.3 (0.5)  16.0 (1.8) |
| **Intervention fidelity (heart rate data)**^3^  Average HR during sessions, mean beats per minute (SD)  Average HR during sessions, mean % of HRmax (SD)  Peak HR during sessions, mean beats per minute (SD)  Peak HR during sessions, mean % of HRmax (SD) | 133.9 (13.1)  65.6 (6.6)  155.6 (13.6)  76.2 (6.5) |
| **Acceptability (teacher reported)**^4^  Overall, mean (SD)  Professional learning, mean (SD)  Support from research team, mean (SD) | 4.7 (0.6)  4.6 (0.6)  4.6 (0.7) |
| **Appropriateness (teacher reported)**^4^  Student enjoyment, mean (SD)  Student health and wellbeing, mean (SD)  Student behavior in the classroom, mean (SD) | 4.1 (0.8)  4.2 (0.7)  4.1 (0.5) |
| **Sustainment (teacher reported)**^4^ |  |
| Intention to deliver the program in the future, mean (SD) | 4.0 (0.8) |

*Note.* B2La = Burn 2 Learn adapted; SAAFE = Supportive, Active, Autonomous, Fair and Enjoyable; HR = heart rate; SD = standard deviation; BPM = beats per minute

^1^Teachers asked to record the number of B2La sessions they delivered in Phases 1 to 3 in the B2La Teacher Handbook.

^2^ Observations of session quality of scored on a 4-point Likert scale- Strongly disagree (1) to Strongly agree (4), total /20.

^3^ Mean peak heart rate (% HRmax) and mean heart rate for the entire session (% HRmax) extracted from the B2L app.

^4^ Scored on a 5-point Likert scale ranging from Strongly disagree (1) to Strongly agree (5)

**Supplementary Figure 1. Study timeline**

**
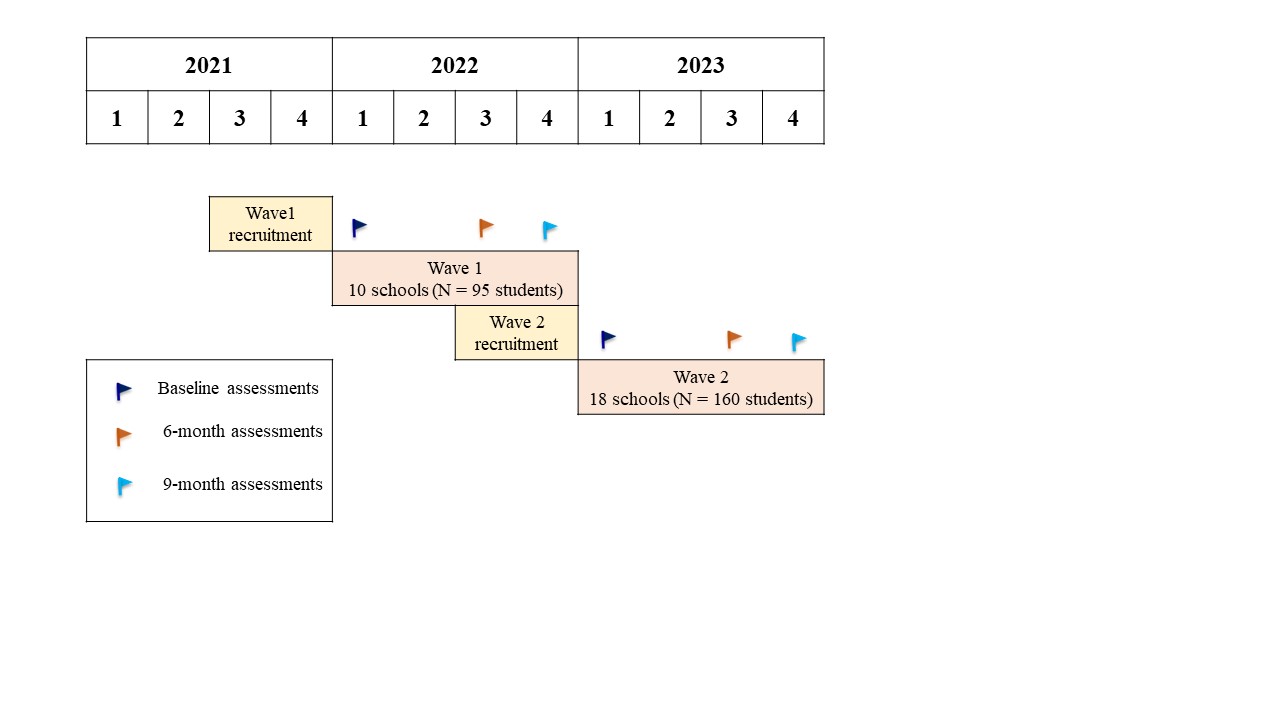
**

**Supplementary Figure 2. B2La implementation framework**


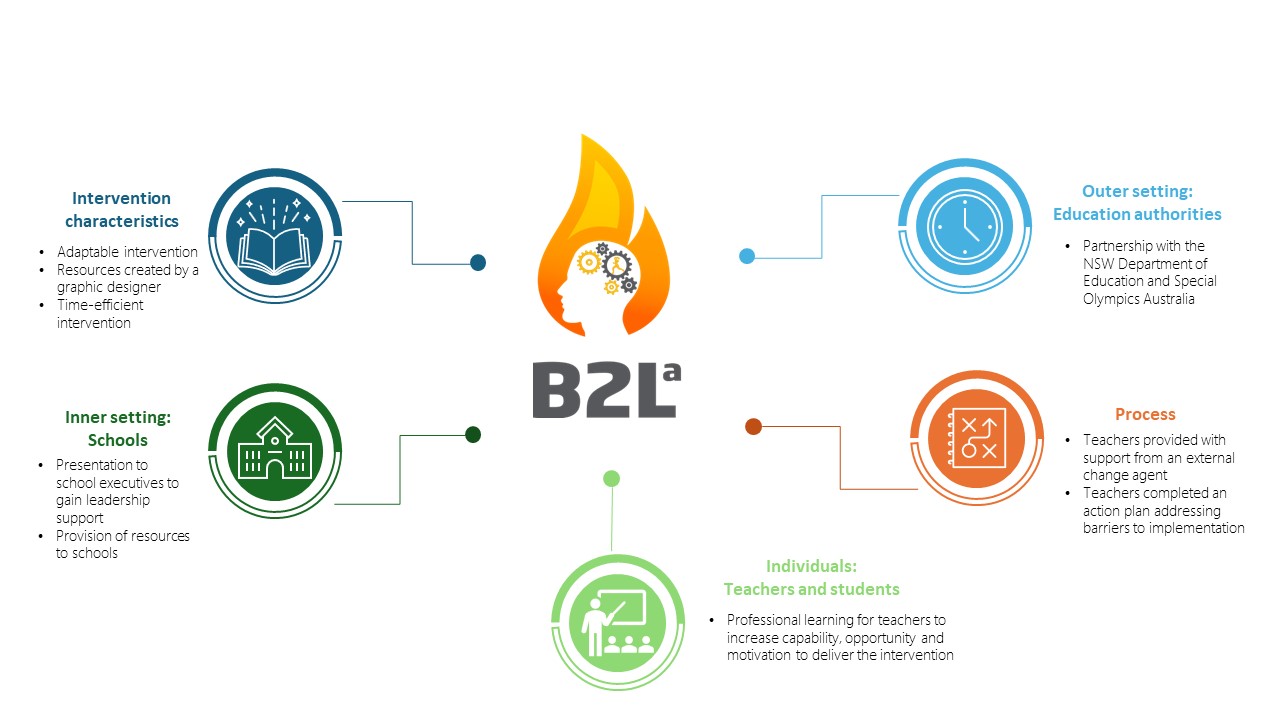

Supplement: Supplementary file 2 — Supplementary Material 2. [file 12966_2025_1829_MOESM2_ESM.docx]
